# The Obesity-Related Dietary Pattern Is Associated with Higher Risk of Sleep Disorders: A Cross-Sectional Study from NHANES

**DOI:** 10.3390/nu14193987

**Published:** 2022-09-26

**Authors:** Shanze Wang, Chaonan Fan, Yingying Zhu, Xijia Tang, Li Ling

**Affiliations:** 1 Department of Medical Statistics, School of Public Health, Sun Yat-sen University, Guangzhou 510080, China; 2 Clinical Research Design Division, Clinical Research Center, Sun Yat-sen Memorial Hospital, Sun Yat-sen University, Guangzhou 510120, China

**Keywords:** sleep disorders, dietary pattern, obesity, partial least squares

## Abstract

Evidence on the association between dietary patterns and sleep disorders is limited and controversial. In addition, studies evaluating the effect of dietary patterns on sleep disorders have seldom considered the critical role of obesity. We aimed to explore obesity-related dietary patterns and evaluate their impact on sleep disorders using data from the National Health and Nutrition Examination Survey 2005–2014. In total, 19,892 participants aged over 20 years with two-day dietary recalls were enrolled. Obesity-related dietary patterns explaining most variance in waist circumference and BMI simultaneously were extracted from twenty-six food groups by the using partial least squares method. Sleep disorder and sleep duration, which were defined by self-reported questions, were the primary and the secondary outcome, respectively. Generalized linear models were performed to estimate the association of sleep disorders and sleep duration with dietary patterns. Two types of dietary patterns were identified. The “high fats, refined grains, and meat” pattern was characterized by high intakes of solid fats, cured meat, potatoes, refined grains, meat, cheese, and added sugars. The “low whole grains, vegetables, and fruits” pattern was characterized by low intakes of oils, whole grains, nuts and seeds, milk, fruits, and several vegetables. Participants with the highest adherence to the “high fats, refined grains, and meat” pattern had a higher risk for sleep disorders (OR (95%CI): 1.43 (1.12, 1.84)) and shorter sleep duration (β (95%CI): −0.17 (−0.26, −0.08)) compared to those with the lowest adherence. The corresponding associations for the “low whole grains, vegetables, and fruits” pattern were only significant for sleep duration (β (95%CI): −0.26 (−0.37, −0.15)). Our results found that the dietary pattern characterized by high solid fats, cured meat, potatoes, refined grains, meat, cheese, and added sugars, was associated with a higher risk for sleep disorders and shorter sleep duration.

## 1. Introduction

Sleep disorders refer to a series of symptoms that disturb individuals’ sleep patterns, duration, and quality, among which insomnia, sleep apnea, and restless legs syndrome are the most common ones [1]. Sleep disorders affect 50 to 70 million Americans [2] and are associated with varieties of adverse health outcomes, including type 2 diabetes [3], cardiovascular diseases [4,5], and all-cause mortality [6]. Preventive strategies are therefore warranted to tackle sleep disorders and to reduce their threat to public health.

Previous studies have examined the contribution of dietary patterns to the risk of developing chronic non-communicable diseases [7,8], but evidence on the association between dietary patterns and sleep disorders is limited and inconsistent. For example, several studies had shown that better adherence to healthy dietary patterns characterized by high fruits, vegetables, whole grains, and high-quality proteins, including Healthy Eating Index-2015 [9], Mediterranean Diet [10], Dietary Approach to Stop Hypertension [11], and other exploratory dietary patterns was inversely associated with sleep disorders [12,13,14]. In addition, a dietary pattern high in sugar-sweetened beverages, fast foods, and bread was associated with a higher risk of sleep disorders in Mexican adults [12], while other studies found protective or no statistically significant associations between dietary patterns sharing similar characteristics and sleep quality [15,16,17].

Besides, considerable evidence supported that there was a strong association between obesity and sleep disorders [18,19,20,21,22]. The potential pathogenic causes might be the alteration of upper airway structure and function, chronic emotional stress, inflammatory burden, and obesity-related comorbidities that have negative effects on sleep quality [18,22]. In addition, published studies had shown the associations between dietary patterns and weight change [23,24,25]. Obesity appeared to be on the pathway between dietary patterns and sleep disorders. Therefore, information about obesity should be incorporated in extracting dietary patterns instead of being confounding factors to be adjusted in previous studies [14,15].

Partial least squares (PLS) takes prior knowledge about intermediate response variables related to the health or disease outcome into account when deriving dietary patterns, and those dietary patterns are more specific and stronger in evaluating diet–disease association [26,27]. Previous study has incorporated information about obesity to extract obesity-related dietary patterns that explained the most variability in obesity by PLS [27]. Nevertheless, no study has evaluated the relationship between obesity-related dietary pattern and sleep disorders.

In this study, we aimed to derive obesity-related dietary patterns among American adults with PLS and further examined the association between dietary patterns and sleep disorders. Moreover, sleep disorders may reduce sleep duration, as they cause frequent awakenings and disruption of sleep [28,29]. Thus, the association of sleep duration with dietary patterns was examined to support findings for sleep disorders. An understanding of dietary patterns in relation to obesity will provide supplementary evidence for dietary recommendations that are beneficial to the prevention of sleep disorders.

## 2. Materials and Methods

### 2.1. Data and Study Population

The National Health and Nutrition Examination Surveys (NHANES) are a series of cross-sectional population-based surveys utilizing a complex, multistage, probability sampling design to create a representative sample of the civilian, non-institutionalized US population. The survey examines about 5000 persons each year and the data are released in two-year cycles to the public. Detailed information on data-collection procedures and analytic guidelines for NHANES datasets has been provided elsewhere [30,31]. Questionnaires containing information about sleep disorders were incorporated from 2005 to 2014, and a total of five cycles (NHANES 2005–2014) were used for this study.

There were 22,572 participants aged over 20 years with complete and reliable two-day 24-h dietary recall data. A total of 2680 participants were excluded for the following reasons: (i) no information about sleep disorders and sleep duration (*n* = 66); (ii) implausible dietary intake (the average of two-day dietary energy intake < 500 kcal/day or > 5000 kcal/day, *n* = 198); (iii) with missing measures on any of response variables for PLS (*n* = 850); and (iv) with missing measures on any of the covariates for generalize linear models (*n* = 2451). Finally, 19,892 participants defined the final analytical sample.

### 2.2. Definition of Outcome

In this study, the participant with a diagnosed sleep disorder was defined by an affirmative answer to the question “Have you ever been told by a doctor or other health professional that you have a sleep disorder?” Information on sleep duration is derived from responses to the question “How much sleep do you usually get at night on weekdays or workdays?” Reported sleep duration of ≥12 h was coded as 12 in NHANES datasets.

### 2.3. Dietary Assessment

NHANES uses 24-h dietary recall to collect dietary data on the types and amounts of foods and beverages Americans report that they consume. The Food Patterns Equivalents Database (FPED) then converts the dietary data to the respective number of cup/ounce/teaspoon equivalents of food groups defined according to the latest update of the Dietary Guidelines for Americans [32]. Twenty-six food groups were used (fruits, fruit juice, dark green vegetables, tomatoes, other red and orange vegetables, potatoes, other starchy vegetables, other vegetables, beans and peas, whole grains, refined grains, meat, cured meat, organ meat, seafood, poultry, eggs, soybean products, nut and seeds, milk, yogurt, cheese, oil, solid fats, added sugars, and alcoholic drinks) to derive dietary patterns [32].

### 2.4. Covariates

Sociodemographic and lifestyle characteristics were deemed as covariates in our analysis referring to published studies [10,13,14]. Sociodemographic characteristics included age (year: 20–39, 40–59, 60+), gender, race (Mexican American, Other Hispanic, Non-Hispanic White, Non-Hispanic Black, and Other Race), educational level (≤11th grade, high school grade or equivalent, and college or above), poverty income ratio (PIR) (≤130%, 131–185%, and ≥186%) [33], and employment status (employment or unemployment). Lifestyle characteristics included physical activity calculated from the Physical Activity questionnaire using metabolic equivalents (MET) minutes per week (<600 MET-min/week, ≥600 MET-min/week) [34], smoking status (never, ever, and current), and energy intake using the average of two-day dietary energy intake (kcal/day).

### 2.5. Statistical Analysis

All statistical analyses accounted for the complex sample survey design of NHANES. The dietary two-day sample weight was used and variance estimation was calculated by the Taylor series linearization method as recommended by the NHANES [35].

#### 2.5.1. Extraction of the Obesity-Related Dietary Patterns

In this study, PLS was used to derive dietary patterns in relation to obesity. PLS takes prior knowledge about intermediary response variables related to the health or disease outcome into account and maximizes the covariance between linear combinations of predictors and responses in driving dietary patterns [26]. Dietary patterns identified by PLS are more specific and stronger in evaluating diet–disease association than principal component analysis maximizing explained variance in predictors [36]. Nutrients, biomarkers, or determinants on the pathway to a particular health outcome, were typically selected as response variables [26,36,37]. In this study, we chose waist circumference and body mass index (BMI) as response variables to derive obesity-related dietary patterns. Predictor variables were the 26 predefined food groups from FPED. To avoid the influence of the different units, both predictor variables and response variables were standardized before deriving dietary patterns. The number of extracted dietary patterns was determined by the minimum predicted residual sum of squares (PRESS) using random-sample cross-validation and patterns’ interpretability [36]. For naming and interpreting dietary patterns, food groups with absolute values of factor loadings ≥0.20 were considered to contribute significantly to the corresponding dietary pattern [38]. Dietary pattern scores were calculated by summing the food group intake weighted by the corresponding factor loading across all 26 food groups. The participants were divided into four groups (Q1–Q4) for further analysis according to quartiles of the dietary pattern score in each dietary pattern and the Q4 group represented the highest adherence to the corresponding pattern.

#### 2.5.2. Descriptive Analysis and Modeling

Characteristics of participants were presented as the weighted mean and weighted standard deviation for continuous variables as well as sample counts and weighted percentages for categorical ones. Differences between participants with and without sleep disorders were tested by Rao–Scott Chi-square for categorical variables and independent t-tests adjusted for sample weights for continuous variables. Kruskal–Wallis tests adjusted for sample weights were used for comparison among quartiles in each dietary pattern. The weighted multivariable logistic regression model was applied to assess the relationship between each quartile of dietary pattern scores and the risk of sleep disorders with the lowest quartile used as the reference category. Model 1 was adjusted for age, gender, and energy intake. Model 2 was additionally adjusted for race, education level, PIR, employment status, smoking, and physical activity. We further tested linear trends for categorical variables by assigning the median dietary pattern score to each quartile of each dietary pattern, generating a continuous variable in the models. In addition, sleep duration was used as a secondary outcome and its association with dietary patterns was explored using weighted multivariable linear regression model to support our findings on sleep disorders.

Sensitivity analysis was performed to test whether misreporting of energy intake would affect the relation of dietary patterns to sleep disorders, as misreporting of energy intake in nutritional surveys may undermine the valid estimation of diet–disease relationships. We classified each participant as under-reporter, plausible reporter, or over-reporter according to the ratio of energy intake to estimated energy requirement (EER) and additionally adjusted for the reporting groups to control the effect of misreporting as suggested by Jessri et al. [39]. Cutoffs for the categorization were defined using the 95% confidence limits of the expected ratio of 1. EER was calculated using the equations developed by the Institute of Medicine (IOM), based on age, height, weight, and physical activity level (PAL) [40]. Methodology about the derivation of participants’ PAL from physical activities measured by MET has been published elsewhere [41]. Based on NHANES 2005–2014 data, under-reporters, plausible reporters, and over-reporters were classified as having the ratio of energy intake to EER < 0.61, 0.61–1.39, and >1.39, respectively.

Significance was set as two-sided *p* < 0.05. All analyses in this study were performed by R version 4.1.2 with pls and survey package.

## 3. Results

### 3.1. Study Population

Totally, 19,892 participants from NHANES 2005–2014 with a mean age of 46.8 years and 52.5% female were included in this study. Among all participants, 8.5% (1671/19,892) participants reported having sleep disorders. Compared with those without sleep disorders, participants with sleep disorders were more likely to be older, unemployed, lacked physical activities, and had low income (all *p* < 0.05). Besides, participants with sleep disorders had significantly higher waist circumference and BMI (both *p* < 0.001). The energy intake was 2092.9 kcal/day and no significant difference was found between the two groups (Table 1).

### 3.2. Obesity-Related Dietary Patterns

**Figure 1:**
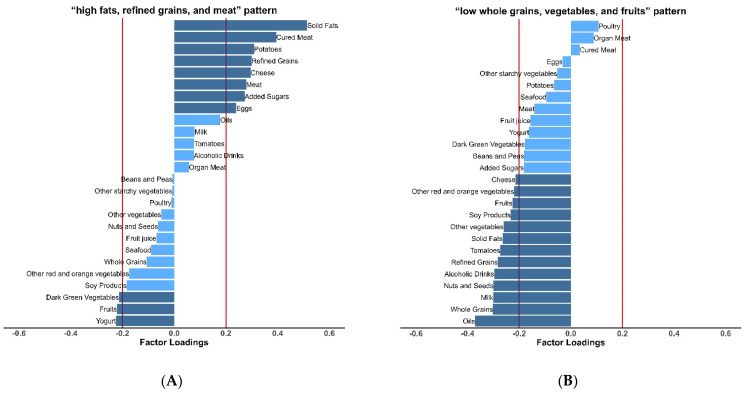
Factor loadings from partial least squares of food group intakes. The first dietary pattern was labeled the “high fats, refined grains, and meat” pattern (**A**). The second dietary pattern was labeled the “low whole grains, vegetables, and fruits” pattern (**B**). The darker color indicates that food groups with absolute values of factor loadings ≥ 0.20.

The absolute minimum PRESS criterion was met with three dietary patterns (Figure S1). The third dietary pattern had little improvement on PRESS with low explained variance in food groups and response variables. Thus, the third dietary pattern was removed and the first two were identified as obesity-related dietary patterns for further analyses. Solid fats, cured meat, potatoes, refined grains, meat, cheese, and added sugars had positive factor loadings on the first dietary pattern, which mainly represented a “high fat, refined grains, and meat” pattern. The second dietary pattern, characterized by low intakes of oils, whole grains, nuts and seeds, milk, fruits, and several vegetables, was labeled as the “low whole grains, vegetables, and fruits” pattern (Figure 1). Two dietary patterns together explained 16.30% of the variance in food groups and explained 2.66% and 2.43% of the variance in waist circumference and BMI, respectively (Table S1).

Compared to participants with lower scores in the “high fats, refined grains, and meat” pattern, those with higher scores were younger, more likely to be male, current smokers, and unemployed, with lower education level, physical activities, and had higher energy intake (all *p* < 0.05). Participants adhering to the “low whole grains, vegetables, and fruits” pattern were older, mostly female, less educated, and unemployed, and had lower PIR, physical activities, and energy intake (all *p* < 0.05). For anthropometric characteristics, both dietary pattern scores were positively associated with waist circumference and BMI (all *p* < 0.05, Tables S2 and S3).

In addition, for factors related to anthropometric characteristics, participants with the highest adherence to the “high fats, refined grains, and meat” pattern were more likely to consider themselves underweight and less likely to lose weight (all *p* < 0.05). With regard to the “low whole grains, vegetables, and fruits” pattern, participants in the highest quartile (Q4) were more likely to consider themselves overweight and to lose weight, especially by eating less, compared with those in the first quartile (Q1, all *p* < 0.05, Table S4).

### 3.3. Dietary Patterns and Sleep Disorders

There were significant differences in the prevalence of sleep disorders among quartiles of the “high fats, refined grains, and meat” pattern. Compared with participants in Q1, those in Q4 had a higher risk (OR (95%CI): 1.41 (1.12, 1.77), *p* = 0.005) of having sleep disorders in the crude model. The association was strengthened when age, gender, and energy intake were further adjusted in Model 1 (OR (95%CI): 1.66 (1.31, 2.11), *p* < 0.001). Adjusting for all potential confounders did not change the association above (OR (95%CI): 1.43 (1.12, 1.84), *p* = 0.007). Increasingly elevated ORs were along with higher adherence to the “high fats, refined grains, and meat” pattern in all models (all *p* for trend < 0.05). There was no significant association found between the “low whole grains, vegetables, and fruits” pattern and sleep disorders except for Model 1. Participants in Q4 had a higher risk (OR (95%CI): 1.33 (1.05, 1.68), *p* = 0.019) of having sleep disorders relative to Q1 after controlling for age, gender, and energy intake (Table 2).

### 3.4. Dietary Patterns and Sleep Duration

For both dietary patterns, compared with participants in the lowest quartile, those in the highest quartile were associated with shorter sleep duration in the crude model (β (95%CI) for “high fats, refined grains, and meat” pattern: −0.21 (−0.29, −0.13); “low whole grains, vegetables, and fruits” pattern: −0.21 (−0.29, −0.12); all *p* < 0.001). After adjusting for all covariates, the association between two dietary patterns with sleep duration remained (β (95%CI) for “high fats, refined grains, and meat” pattern: −0.17 (−0.26, −0.08); “low whole grains, vegetables, and fruits” pattern: −0.26 (−0.37, −0.15); all *p* < 0.001). Reduction of sleep duration was along with higher adherence to both dietary patterns in all models (all *p* for trend < 0.05, Table 3).

### 3.5. Sensitivity Analysis

In sensitivity analysis, additionally adjusting for the reporting groups did not change the majority of our results. Participants in Q4 of the “high fats, refined grains, and meat” pattern still had a higher risk (OR (95%CI): 1.42 (1.10–1.85), *p* = 0.010) of having sleep disorders relative to Q1, and linear trends for “high fats, refined grains, and meat” pattern remained (*p* for trend = 0.013, Table S5). In addition, the associations and linear trends between dietary patterns and sleep duration were similar to our main results (Table S6).

## 4. Discussion

In this nationally representative survey of American adults, among 19,892 participants, 8.5% (sample *n* = 1671) of total reported having sleep disorders. Two dietary patterns, which explained maximum variance in food groups and obesity, were identified. Adherence to the “high fats, refined grains, and meat” pattern, characterized by high solid fats, cured meat, potatoes, refined grains, meat, cheese, and added sugars was associated with a higher risk of sleep disorders, and these associations were dose-dependent. No significant association between the “low whole grains, vegetables, and fruits” pattern, characterized by a low intake of oils, whole grains, nuts and seeds, milk, fruits, and several vegetables, and sleep disorders was found after all potential covariates were adjusted. Both dietary patterns were associated with a short duration of sleep.

The positive association of the “high fats, refined grains, and meat” pattern with sleep disorders was possibly explained by the high proportion of food with high saturated fats, glycemic index (GI), and unfavorable proteins from meat. Our results were in line with previous studies demonstrating high saturated fat consumption increases the risk of insomnia and daytime sleepiness [42,43]. However, studies on the effect of carbohydrates yielded controversial results. A dietary pattern based on fast food, sweets, and fizzy drinks was significantly associated with higher sleep duration [15] and carbohydrate-based high-GI diets resulted in a significant shortening of sleep latency [44]. In contrast, cross-sectional studies reported that a dietary pattern high in sugar-sweetened beverages, bakery products, and fast foods is associated with high risk of insomnia, and a low intake of rice but high intakes of wheat and other grains as staple food could be protective against insomnia in Mexico and China, respectively [12,14]. Recent results from a prospective cohort study also concluded that high-GI diets are a risk factor for insomnia in postmenopausal women [45]. Potential mechanisms may be hyperglycemia and the release disorder of autonomic counterregulatory hormones induced by high-GI diets, which may contribute to sleep disorders [46]. As potatoes, refined grains, and added sugars all have a high GI [47], adherence to the “high fats, refined grains, and meat” pattern may have negative impacts on sleep disorders. Further research is required to explore the mechanistic aspect of the relationship between high-GI diets and sleep disorders. In the current study, meat consumption, mainly comprised of processed meat and red meat, was another major constituent of the “high fats, refined grains, and meat” pattern, particularly when we sum the factor loadings of cured meat and meat. High consumption of processed meat and red meat may lead to unfavorable antioxidant status and increased levels of inflammatory biomarkers [48,49,50], which had presented a bidirectional relationship with sleep apnea and restless legs syndrome [51,52]. Moreover, a randomized controlled trial conducted on 49 overweight or obese men aged 30–65 years with chronic insomnia showed that diet intervention with recommended nutrient composition could reduce sleep onset latency [53]. These results remind us that the synergistic effect of solid fats, cured meat, refined grains, meat, and added sugars might increase the risk of sleep disorders.

Moreover, we found that waist circumference and BMI increased with increasing adherence to the “high fats, refined grains, and meat” pattern. Saturated fats, refined grains, added sugar, and processed meats were all major components in dietary patterns that were associated with obesity [54,55,56]. Excess weight in turn can elevate the risk of insomnia [18,19]. Therefore, improving diet may be warranted to minimize the deleterious impact of both diet and excess weight induced by diet on sleep health, given the intakes of solid fats and added sugars are still above recommended levels and the high prevalence of obesity among Americans [57,58].

Compared to low adherence, a higher risk of sleep disorders was found in participants with the highest adherence to the “low whole grains, vegetables, and fruits” pattern after adjusting for age, gender, and energy intake. Due to the high inter-correlation of diet with energy intake, failure to account for energy intake in epidemiologic studies may obscure or reverse diet–disease associations [59]. In this study, stronger associations were found after adjustment for energy intake in both dietary patterns. Multiple epidemiological studies had also concluded that dietary patterns with high nutrient-dense foods such as whole grains, vegetables, or fruits are protective factors against insomnia, sleep apnea and daytime sleepiness [11,14,60]. In the present study, participants following the “low whole grains, vegetables, and fruits” pattern also have a low intake of solid fats and refine grains, which may partially account for the inverse association between the “low whole grains, vegetables, and fruits” pattern and sleep disorders but without statistical significance, after all potential covariates were adjusted.

As the dietary pattern score of the “low whole grains, vegetables, and fruits” pattern increased, participants tended to consume less energy but with increased waist circumference and BMI. In our study, participants following the “low whole grains, vegetables, and fruits” pattern were more likely to weigh less, considered themselves to be overweight, and tend to eat less to lose weight. This might explain participants with higher waist circumference and BMI had lower energy intake. Besides, in nutritional epidemiology studies, energy underreporting is associated with both obesity and current weight loss attempts, especially in 24-h dietary recall [61,62].

For both dietary patterns, higher adherence to dietary patterns was associated with shorter sleep duration. Shorter sleep duration is associated with sleep disorders. Possible mechanisms may be frequent awakenings and disruption of sleep caused by sleep disorders (e.g., restless leg syndrome, sleep apnea), which could further lead to a short duration of sleep [28,29]. In addition, previous studies have shown that short sleep duration may represent a marker for sleep disorder severity [63]. The present results corroborated our findings that the “high fats, refined grains, and meat” pattern was deleterious to sleep health. For the “low whole grains, vegetables, and fruits” pattern, although no significant association was found with sleep disorders, the sleep duration of participants in Q3 and Q4 was lower than the recommended minimum level (7 h), which would influence many aspects of health [64].

Our study had several strengths. First, to our knowledge, this is the first study using a nationally representative study to examine obesity-related dietary patterns extracted by PLS among the American population concerning sleep disorders. Prior knowledge that obesity has a deleterious effect on sleep wellness and is highly correlated with dietary intake could be incorporated into the dietary pattern analysis, which would discover novel disease-specific dietary patterns and better illustrate the effect of overall diet on sleep disorders. Furthermore, the large sample size with sample weights incorporated in all algorithms allowed us to make estimates that are representative and have sufficient statistical reliability. Lastly, sensitivity analysis by taking account of misreporting showed that the majority of our results were robust.

The findings of the present study should be considered in light of several limitations. First of all, due to the cross-sectional study design of NHANES, causality or the direction of the relationship is limited and residual confounding could still be possible although we adjusted for a great number of covariates. Second, data on food intake were from 24-h dietary recall, which relies on the participants’ memory and is therefore prone to recall bias. However, NHANES uses an automated multiple-pass method to optimize dietary data collection [65]. Besides, to improve the accuracy of dietary assessment, we only enrolled participants with complete and reliable two-day dietary recall data. Third, the definition of diagnosed sleep disorders solely relied on a self-reported question and did not provide a definition or examples of sleep disorders for the participants, which may lead to misreporting because of a lack of objective measurement.

## 5. Conclusions

In conclusion, using data from a nationally representative survey, we incorporated prior knowledge of obesity in deriving dietary patterns and found that a dietary pattern characterized by high solid fats, cured meat, potatoes, refined grains, meat, cheese, and added sugars was associated with higher risk for sleep disorders and shorter sleep duration among American adults. Evidence from this study can help increase awareness of the effect of obesity-related dietary patterns on sleep disorders and provide supplementary advice for dietary recommendations that are protective against sleep disorders.

## Figures and Tables

**Table 1:** Characteristics of study participants with and without sleep disorders, NHANES 2005–2014 ^1^.

	Total	Sleep Disorders	Without Sleep Disorders	*p* Value ^4^
*n*	19,892	1671	18,221	
**Age, %**				<0.001
20–39	6824 (37.5)	335 (21.6)	6489 (39.0)	
40–59	6651 (38.1)	711 (50.1)	5940 (37.0)	
60+	6417 (24.4)	625 (28.3)	5792 (24.0)	
**Age (year)** ^2^	46.8 (16.7)	51.0 (14.5)	46.4 (16.8)	<0.001
**Female, %**	10,432 (52.5)	814 (48.4)	9618 (52.9)	0.014
**Race, %**				<0.001
MA	2987 (8.1)	160 (5.0)	2827 (8.4)	
OH	1647 (4.7)	154 (4.8)	1493 (4.7)	
NHW	9533 (69.8)	918 (74.7)	8615 (69.4)	
NHB	4117 (10.9)	350 (10.9)	3767 (10.9)	
OR	1608 (6.4)	89 (4.6)	1519 (6.6)	
**Education level, %**				0.108
≤11th grade	4708 (15.8)	356 (14.0)	4352 (16.0)	
High school grade orequivalent	4549 (22.6)	405 (24.8)	4144 (22.4)	
College or above	10,635 (61.6)	910 (61.2)	9725 (61.6)	
**Poverty income ratio, %**				0.029
≤130%	6091 (21.3)	585 (24.0)	5506 (21.1)	
131–185%	2556 (10.5)	226 (12.4)	2330 (10.3)	
≥186%	11,245 (68.2)	860 (63.6)	10,385 (68.6)	
**Unemployment, %**	8638 (36.4)	939 (45.4)	7699 (35.5)	<0.001
**Smoking, %**				<0.001
Current	4014 (20.4)	406 (24.5)	3608 (20.0)	
Ever	5006 (24.6)	523 (30.0)	4483 (24.1)	
Never	10,872 (55.0)	742 (45.5)	10,130 (55.8)	
**Physical activity ≥ 600 MET-min/week, %**	6754 (39.0)	445 (29.3)	6309 (39.9)	<0.001
**Sleep duration (hours)** ^2^	6.9 (1.3)	6.5 (1.6)	7.0 (1.3)	<0.001
**Energy intake (kcal/day)** ^2, 3^	2092.9 (765.6)	2073.7 (776.0)	2094.6 (764.7)	0.511
**Waist circumference (cm)** ^2^	98.6 (16.4)	108.5 (18.7)	97.7 (15.9)	<0.001
**BMI (kg/m^2^)** ^2^	28.8 (6.7)	32.5 (7.9)	28.5 (6.5)	<0.001

^1^ All results were survey-weighted except for counts of categorical variables; ^2^ Mean (SD); ^3^ Energy intake was the average energy intake from two-day dietary recalls; ^4^ *p* value obtained from Rao–Scott Chi-square tests for categorical variables and independent t-tests tests adjusted for sample weights for continuous variables. MA, Mexican American; OH, Other Hispanic; NHW, Non-Hispanic White; NHB, Non-Hispanic Black; OR, Other race; MET, metabolic equivalents; BMI, body mass index.

**Table 2:** Odds ratios of sleep disorders and corresponding 95% CIs according to quartiles of dietary pattern scores from partial least squares ^1^.

	Quartile of Dietary Pattern Scores				
	Q1	Q2	Q3	Q4	*p* for Trend
“high fats, refined grains, and meat” pattern					
Sleep disorder/Total	367/5202	433/5149	412/4863	459/4678	
Crude	1.0 (Ref.)	1.23 (0.97, 1.55)	1.13 (0.91, 1.40)	1.41 (1.12, 1.77) **	0.009
Model 1	1.0 (Ref.)	1.29 (1.02, 1.62) *	1.26 (1.02, 1.56) *	1.66 (1.31, 2.11) ***	<0.001
Model 2	1.0 (Ref.)	1.18 (0.93, 1.51)	1.14 (0.92, 1.40)	1.43 (1.12, 1.84) **	0.009
“low whole grains, vegetables, and fruits” pattern					
Sleep disorder/Total	323/4313	360/4663	454/5177	534/5739	
Crude	1.0 (Ref.)	0.99 (0.82, 1.20)	1.16 (0.93, 1.45)	1.18 (0.96, 1.44)	0.084
Model 1	1.0 (Ref.)	1.03 (0.84, 1.27)	1.25 (0.99, 1.58)	1.33 (1.05, 1.68) *	0.013
Model 2	1.0 (Ref.)	0.99 (0.80, 1.22)	1.18 (0.92, 1.51)	1.18 (0.92, 1.51)	0.130

^1^ All results were survey-weighted except for sample counts; * *p* < 0.05; ** *p* < 0.01; *** *p* < 0.001. CI, confidence interval; Q, quartiles. Model 1: Crude model additionally adjusted for age, gender, and energy intake; Model 2: Model 1 additionally adjusted for race, education, poverty income ratio, employment status, smoking, and physical activity.

**Table 3:** Coefficients of sleep duration and corresponding 95% CIs according to quartiles of dietary pattern scores from partial least squares ^1^.

	Quartile of Dietary Pattern Scores				
	Q1	Q2	Q3	Q4	*p* for Trend
“high fats, refined grains, and meat” pattern					
Crude	1.0 (Ref.)	−0.04 (−0.11, 0.04)	−0.07 (−0.14, 0)	−0.21 (−0.29, −0.13) ***	<0.001
Model 1	1.0 (Ref.)	−0.04 (−0.12, 0.03)	−0.08 (−0.15, 0) *	−0.23 (−0.31, −0.15) ***	<0.001
Model 2	1.0 (Ref.)	−0.02 (−0.10, 0.06)	−0.04 (−0.11, 0.04)	−0.17 (−0.26, −0.08) ***	0.001
“low whole grains, vegetables, and fruits” pattern					
Crude	1.0 (Ref.)	−0.01 (−0.08, 0.05)	−0.09 (−0.16, −0.02) *	−0.21 (−0.29, −0.12) ***	<0.001
Model 1	1.0 (Ref.)	−0.10 (−0.17, −0.03) **	−0.23 (−0.31, −0.16) ***	−0.40 (−0.51, −0.29) ***	<0.001
Model 2	1.0 (Ref.)	−0.05 (−0.12, 0.02)	−0.15 (−0.23, −0.07) ***	−0.26 (−0.37, −0.15) ***	0.001

^1^ All results were survey-weighted except for sample counts; * *p* < 0.05; ** *p* < 0.01; *** *p* < 0.001. CI, confidence interval; Q, quartiles. Model 1: Crude model additionally adjusted for age, gender, and energy intake; Model 2: Model 1 additionally adjusted for race, education, poverty income ratio, employment status, smoking, and physical activity.

## Data Availability

The NHANES datasets are publicly available online from NHANES, accessible at the NHANES website: https://wwwn.cdc.gov/nchs/nhanes/default.aspx (accessed on 8 December 2021).

## Supplementary Materials

The following supporting information can be downloaded at: https://www.mdpi.com/article/10.3390/nu14193987/s1, Figure S1: PRESS of the different number of factors from PLS in BMI and waist circumference; Table S1: Table of factor loadings and explained variance in food groups and responses in each dietary pattern; Table S2: Characteristics of study participants by quartiles of dietary pattern scores in “high fats, refined grains, and meat” pattern; Table S3: Characteristics of study participants by quartiles of dietary pattern scores in “low whole grains, vegetables, and fruits” pattern; Table S4: Factors related to anthropometric characteristics of study participants by quartiles of dietary factor scores in two dietary patterns; Table S5: Odds ratios of sleep disorders and corresponding 95% Cis according to quartiles of dietary pattern scores from partial least squares adjusted for the reporting groups; Table S6: Coefficients of sleep duration and corresponding 95% Cis according to quartiles of dietary pattern scores from partial least square adjusted for the reporting groups.
