# Supplementary material for: The Obesity-Related Dietary Pattern Is Associated with Higher Risk of Sleep Disorders: A Cross-Sectional Study from NHANES
Source: Nutrients. 2022 Sep 26;14(19):3987. doi: 10.3390/nu14193987 (PMC9572699; doi:10.3390/nu14193987)
Supplement: Supplementary file 1 [file nutrients-14-03987-s001.zip › nutrients-1877420-supplementary.pdf]

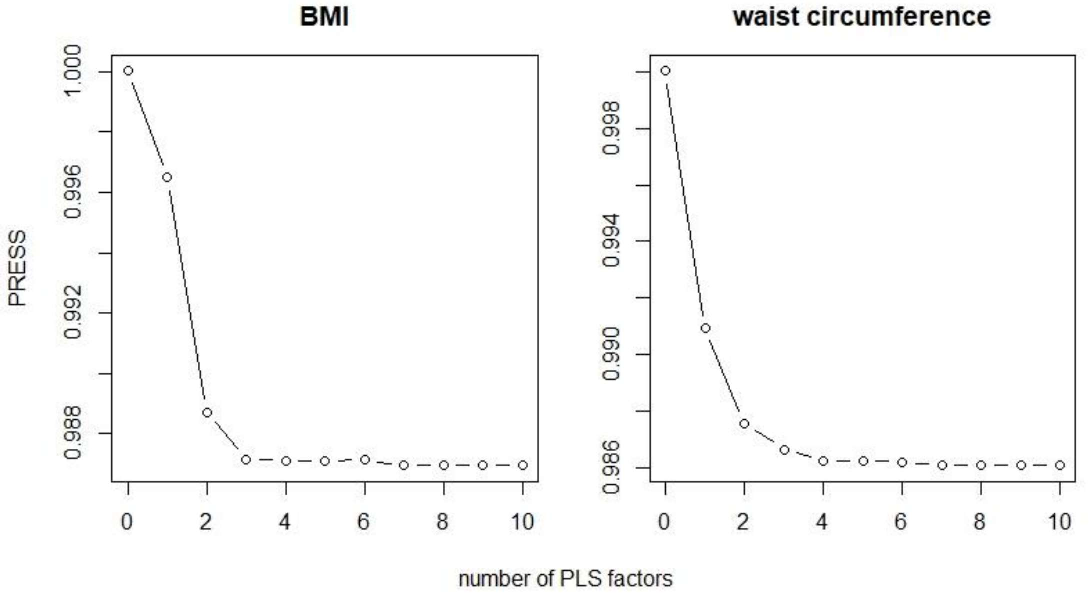

Figure S1. Predicted residual sum of squares (PRESS) of the different number of factors from PLS in BMI and waist circumference.

Table S1. Table of factor loadings and explained variance in food groups and responses in each dietary pattern [1].

Food Groups	"high fats, refined grains, and meat" pattern	"low whole grains, vegetables, and fruits" pattern
Fruits	**-0.22**	**-0.23**
Fruit juice	-0.07	-0.16
Dark Green Vegetables	**-0.21**	-0.18
Tomatoes	0.08	**-0.27**
Other red and orange vegetables (excludes, tomatoes)	-0.17	**-0.22**
Potatoes (white potatoes)	**0.31**	-0.07
Other starchy vegetables (excludes white potatoes)	-0.01	-0.05
Other vegetables	-0.05	**-0.26**
Beans and Peas	-0.01	-0.18
Whole Grains	-0.11	**-0.30**
Refined Grains	**0.30**	**-0.28**
Meat (beef, veal, pork, lamb, game)	**0.28**	-0.14
Cured meat (frankfurters, sausage, corned beef, cured ham and luncheon meat made from beef, pork, poultry)	**0.39**	0.03
Organ meat (from beef, veal, pork,	0.06	0.09

Food Groups	"high fats, refined grains, and meat" pattern	"low whole grains, vegetables, and fruits" pattern
lamb, game, poultry)		
Seafood	-0.09	-0.09
Poultry (chicken, turkey, other fowl)	-0.01	0.11
Eggs	**0.24**	-0.03
Soybean products (excludes calcium fortified soy milk and mature soybeans)	-0.18	**-0.23**
Nuts and Seeds	-0.06	**-0.30**
Milk (includes calcium fortified soy milk)	0.08	**-0.30**
Yogurt	**-0.22**	-0.16
Cheese	**0.30**	-0.21
Oils	0.18	**-0.37**
Solid Fats	**0.51**	**-0.26**
Added Sugars	**0.27**	-0.18
Alcoholic Drinks	0.08	**-0.30**
% explained variance in food groups	9.01	7.28
% explained variance in waist circumference	1.91	0.74
% explained variance in BMI	0.76	1.67

[1] Absolute values of factor loadings ≥ 0.20 are in bold font.

Table S2. Characteristics of study participants by quartiles of dietary pattern scores in "high fats, refined grains, and meat" pattern [1]

Characteristics	"high fats, refined grains, and meat" pattern				p value [4]
	Q1	Q2	Q3	Q4	
**Age, %**					<0.001
20-39	1443 (30.9)	1613 (35.6)	1834 (41.4)	1934 (42.2)	
40-59	1771 (39.6)	1647 (36.6)	1546 (35.9)	1687 (40.4)	
60+	1988 (29.5)	1889 (27.8)	1483 (22.6)	1057 (17.4)	
**Age (year)** [2]	49.6 (16.7)	47.9 (17.3)	45.6 (16.7)	44.1 (15.4)	<0.001
**Female, %**	3528 (70.2)	3186 (63.8)	2375 (49.1)	1343 (27.0)	<0.001
**Race, %**					0.003
MA	737 (7.4)	853 (8.3)	781 (9.1)	616 (7.7)	
OH	539 (5.7)	454 (5.0)	363 (4.1)	291 (4.0)	
NHW	2178 (66.4)	2393 (69.5)	2452 (70.8)	2510 (72.8)	
NHB	986 (9.9)	1077 (10.8)	997 (11.1)	1057 (11.8)	
OR	762 (10.5)	372 (6.4)	270 (4.9)	204 (3.7)	
**Education level, %**					<0.001

Characteristics	"high fats, refined grains, and meat" pattern				p value [4]
	Q1	Q2	Q3	Q4	
≤11th grade	1152 (13.6)	1274 (16.1)	1176 (16.6)	1106 (17.1)	
High school grade or equivalent	903 (16.4)	1170 (22.9)	1209 (24.2)	1267 (26.8)	
College or above	3147 (70.1)	2705 (61.0)	2478 (59.2)	2305 (56.1)	
**Poverty income ratio, %**					0.001
≤ 130%	1410 (18.7)	1600 (22.6)	1572 (22.4)	1509 (21.6)	
131% - 185%	661 (9.8)	703 (11.3)	614 (11.0)	578 (9.7)	
≥ 186%	3131 (71.5)	2846 (66.1)	2677 (66.6)	2591 (68.6)	
**Unemployment, %**	2402 (39.1)	2444 (42.4)	2068 (34.0)	1724 (29.9)	<0.001
**Smoking, %**					<0.001
Current	576 (11.5)	927 (19.2)	1157 (24.3)	1354 (26.7)	
Ever	1277 (24.3)	1313 (25.8)	1208 (23.7)	1208 (24.7)	
Never	3349 (64.2)	2909 (55.0)	2498 (52.0)	2116 (48.6)	
**Physical activity ≥ 600 MET-min/week, %**	2063 (46.5)	1698 (39.2)	1493 (36.1)	1500 (34.0)	<0.001
**Sleep duration (hours)** [2]	7.0 (1.3)	6.9 (1.4)	6.9 (1.3)	6.8 (1.3)	<0.001
**Energy intake (kcal/day)** [2, 3]	1666.12 (595.7)	1795.5 (590.7)	2125.5 (605.3)	2784.8 (724.6)	<0.001
**Waist circumference (cm)** [2]	94.8 (15.3)	97.4 (15.8)	99.7 (16.5)	102.4 (17.0)	<0.001
**BMI (kg/m^2)** [2]	27.7 (6.5)	28.5 (6.4)	29.2 (6.9)	29.8 (7.0)	<0.001

[1] All results were survey-weighted except for counts of categorical variables; [2] Mean (SD); [3] Energy intake was the average energy intake from two-day dietary recalls; [4] p value obtained from Kruskal-Wallis tests adjusted for sample weights. MA, Mexican American; OH, Other Hispanic; NHW, Non-Hispanic White; NHB, Non-Hispanic Black; OR, Other race; MET, metabolic equivalents; BMI, body mass index; Q, quartiles.

Table S3. Characteristics of study participants by quartiles of dietary pattern scores in "low whole grains, vegetables, and fruits" pattern [1]

Characteristics	"low whole grains, vegetables, and fruits" pattern				p value [4]
	Q1	Q2	Q3	Q4	
**Age, %**					<0.001
20-39	1692 (40.3)	1601 (36.5)	1710 (35.5)	1821 (37.7)	
40-59	1546 (40.4)	1575 (38.3)	1713 (37.6)	1817 (36.2)	
60+	1075 (19.3)	1487 (25.1)	1754 (26.9)	2101 (26.1)	
**Age (year)** [2]	45.3 (15.6)	47.3 (16.6)	47.7 (17.0)	46.9 (17.3)	<0.001
**Female, %**	1528 (36.9)	2264 (48.6)	3012 (59.7)	3628 (64.9)	<0.001
**Race, %**					<0.001
MA	665 (7.8)	733 (8.3)	797 (8.7)	792 (7.7)	
OH	273 (3.3)	368 (4.3)	473 (5.3)	533 (6.0)	
NHW	2415 (77.2)	2370 (72.2)	2440 (68.5)	2308 (61.6)	
NHB	537 (5.5)	806 (8.8)	1043 (10.8)	1731 (18.5)	

Characteristics	"low whole grains, vegetables, and fruits" pattern				p value [4]
	Q1	Q2	Q3	Q4	
OR	423 (6.3)	386 (6.4)	424 (6.7)	375 (6.2)	
**Education level, %**					<0.001
≤11th grade	707 (9.6)	987 (15.1)	1257 (16.1)	1757 (22.5)	
High school grade or equivalent	835 (17.8)	963 (19.8)	1221 (25.1)	1530 (27.6)	
College or above	2771 (72.6)	2713 (65.1)	2699 (58.8)	2452 (49.9)	
**Poverty income ratio, %**					<0.001
≤ 130%	1090 (16.9)	1263 (18.3)	1545 (20.4)	2193 (29.7)	
131% - 185%	449 (8.3)	546 (9.6)	684 (11.1)	877 (12.9)	
≥ 186%	2774 (74.8)	2854 (72.1)	2948 (68.5)	2669 (57.4)	
**Unemployment, %**	1485 (28.6)	1879 (34.1)	2370 (38.3)	2904 (44.4)	<0.001
**Smoking, %**					0.045
Current	806 (17.6)	844 (19.5)	1031 (19.7)	1333 (24.9)	
Ever	1163 (26.9)	1245 (26.2)	1300 (24.7)	1298 (20.7)	
Never	2344 (55.5)	2574 (54.3)	2846 (55.6)	3108 (54.4)	
**Physical activity ≥ 600 MET-min/week, %**	1965 (50.4)	1684 (39.7)	1574 (35.2)	1531 (30.6)	<0.001
**Sleep duration (hours)** [2]	7.0 (1.20)	7.0 (1.30)	6.9 (1.32)	6.8 (1.51)	<0.001
**Energy intake (kcal/day)** [2, 3]	2811.3 (766.7)	2221.9 (565.0)	1857.5 (484.6)	1480.6 (489.8)	<0.001
**Waist circumference (cm)** [2]	96.64 (15.5)	98.43 (16.2)	98.6 (16.2)	100.6 (17.4)	<0.001
**BMI (kg/m^2)** [2]	27.6 (6.0)	28.6 (6.4)	29.0 (6.7)	30.1 (7.5)	<0.001

[1] All results were survey-weighted except for counts of categorical variables; [2] Mean (SD); [3] Energy intake was the average energy intake from two-day dietary recalls; [4] p value obtained from Kruskal-Wallis tests adjusted for sample weights. MA, Mexican American; OH, Other Hispanic; NHW, Non-Hispanic White; NHB, Non-Hispanic Black; OR, Other race; MET, metabolic equivalents; BMI, body mass index; Q, quartiles.

Table S4. Factors related to anthropometric characteristics of study participants by quartiles of dietary factor scores in two dietary patterns [1,2].

	Q1	Q2	Q3	Q4	p value [3]
**"high fats, refined grains, and meat" pattern**					
**Tried to lose weight in past year, %**	1863 (45.1)	1781 (43.4)	1472 (37.4)	1232 (32.4)	<0.001
**Consider your weight, %**					<0.001
Overweight	2811 (54.2)	2950 (58.2)	2713 (57.4)	2529 (57.4)	
Underweight	212 (3.9)	217 (3.4)	217 (4.1)	307 (5.7)	
About the right weight	2168 (42.0)	1966 (38.3)	1928 (38.4)	1833 (36.9)	
**Like to weigh more, less or same, %**					<0.001
More	240 (3.9)	271 (4.1)	313 (6.2)	503 (9.6)	
Less	3380 (68.1)	3348 (68.1)	3106 (67.5)	2807 (64.3)	

	Q1	Q2	Q3	Q4	*p* value [3]
Stay about the same	1577 (28.0)	1522 (27.9)	1437 (26.3)	1364 (26.1)	
**Ate less to lose weight, %**	1625 (34.1)	1529 (32.1)	1272 (27.4)	1102 (24.6)	<0.001
**"low whole grains, vegetables, and fruits" pattern**					
**Tried to lose weight in past year, %**	1242 (36.0)	1465 (38.1)	1754 (42.6)	1887 (41.6)	<0.001
**Consider your weight, %**					<0.001
Overweight	2024 (48.3)	2575 (57.0)	3016 (60.2)	3388 (61.7)	
Underweight	232 (4.7)	199 (4.2)	242 (4.1)	280 (4.1)	
About the right weight	2051 (47.0)	1880 (38.8)	1908 (35.6)	2056 (34.2)	
**Like to weigh more, less or same, %**					<0.001
More	375 (8.0)	288 (5.8)	326 (5.5)	338 (4.4)	
Less	2439 (59.7)	2982 (67.8)	3424 (69.6)	3796 (70.9)	
Stay about the same	1499 (32.4)	1385 (26.4)	1421 (24.8)	1595 (24.6)	
**Ate less to lose weight, %**	1059 (26.8)	1236 (27.7)	1521 (32.1)	1712 (31.6)	<0.001

[1] All results are survey-weighted except for sample counts; [2] Missing values were not included; [3] *p* value obtained from Kruskal-Wallis tests adjusted for sample weights. Q, quartiles.

Table S5. Odds ratios of sleep disorders and corresponding 95% CIs according to quartiles of dietary pattern scores from partial least square adjusted for the reporting groups [1].

	Quartile of Dietary Pattern Scores				
	Q1	Q2	Q3	Q4	*p* for Trend
**"high fats, refined grains, and meat" pattern**					
Sleep disorder/Total	367/5202	433/5149	412/4863	459/4678	
Model 1	1.0(Ref.)	1.26 (1.00,1.60)	1.23 (0.99,1.53)	1.66 (1.31,2.11) ***	<0.001
Model 2	1.0(Ref.)	1.31 (1.04, 1.67) *	1.31 (1.05, 1.62) *	1.69 (1.32, 2.16) ***	<0.001
Model 3	1.0(Ref.)	1.21 (0.94, 1.54)	1.17 (0.95, 1.45)	1.42 (1.10, 1.85) *	0.013
**"low whole grains, vegetables, and fruits" pattern**					
Sleep disorder/Total	323/4313	360/4663	454/5177	534/5739	
Model 1	1.0(Ref.)	0.94 (0.77,1.15)	1.06 (0.85,1.32)	0.99 (0.82,1.21)	0.788
Model 2	1.0(Ref.)	1.06 (0.86, 1.31)	1.30 (1.02, 1.65) *	1.30 (1.02, 1.64) *	0.019
Model 3	1.0(Ref.)	1.01 (0.81, 1.25)	1.20 (0.93, 1.56)	1.12 (0.87, 1.43)	0.239

[1] All results were survey-weighted except for sample counts. *p<0.05; **p<0.01; ***p<0.001. CI, confidence interval; Q, quartiles. Model 1: adjusted for the reporting groups; Model 2: Model 1 additionally adjusted for age, gender, and energy intake; Model 3: Model 2 additionally adjusted for race, education, poverty income ratio, employment status, smoking, and physical activity.

Table S6. Coefficients of sleep duration and corresponding 95% CIs according to quartiles of dietary pattern scores from partial least square adjusted for the reporting groups [1].

	Quartile of Dietary Pattern Scores				
	Q1	Q2	Q3	Q4	*p* for Trend
**"high fats, refined grains, and meat" pattern**					
Model 1	1.0(Ref.)	-0.05 (-0.13, 0.03)	-0.10 (-0.17, -0.03) **	-0.26 (-0.34, -0.18) ***	<0.001
Model 2	1.0(Ref.)	-0.05 (-0.13, 0.03)	-0.09 (-0.17, -0.02) *	-0.24 (-0.32, -0.15) ***	<0.001
Model 3	1.0(Ref.)	-0.03 (-0.11, 0.05)	-0.05 (-0.13, 0.03)	-0.18 (-0.27, -0.08) ***	<0.001
**"low whole grains, vegetables, and fruits" pattern**					
Model 1	1.0(Ref.)	-0.04 (-0.11, 0.03)	-0.12 (-0.19, -0.05) **	-0.22 (-0.31, -0.13) ***	<0.001
Model 2	1.0(Ref.)	-0.12 (-0.19, -0.05) **	-0.25 (-0.32, -0.17) ***	-0.39 (-0.49, -0.28) ***	<0.001
Model 3	1.0(Ref.)	-0.07 (-0.13, 0)	-0.16 (-0.25, -0.08) ***	-0.25 (-0.35, -0.14) ***	<0.001

[1] All results were survey-weighted except for sample counts. **p*<0.05; ***p*<0.01; ****p*<0.001. CI, confidence interval; Q, quartiles. Model 1: Crude model additionally adjusted for the reporting groups; Model 2: Model 1 additionally adjusted for age, gender, and energy intake; Model 3: Model 2 additionally adjusted for race, education, poverty income ratio, employment status, smoking, and physical activity;
